# New Supramolecular Hydrogels Based on Diastereomeric Dehydrotripeptide Mixtures for Potential Drug Delivery Applications

**DOI:** 10.3390/gels10100629

**Published:** 2024-09-30

**Authors:** Carlos B. P. Oliveira, André Carvalho, Renato B. Pereira, David M. Pereira, Loic Hilliou, Peter J. Jervis, José A. Martins, Paula M. T. Ferreira

**Affiliations:** 1 Chemistry Centre, School of Sciences, University of Minho, 4710-057 Braga, Portugalid9569@alunos.uminho.pt (A.C.); peterjervis6@gmail.com (P.J.J.); 2 REQUIMTE/LAQV, Laboratório de Farmacognosia, Departamento de Química, Faculdade de Farmácia, Universidade do Porto, R. Jorge Viterbo Ferreira, n228, 4050-313 Porto, Portugal; ren.pereira@gmail.com (R.B.P.); dpereira@ff.up.pt (D.M.P.); 3 Institute for Polymers and Composites, University of Minho, 4800-058 Guimarães, Portugal; loic@dep.uminho.pt

**Keywords:** dehydrotripeptides, diastereomeric mixture, self-assembly, co-assembly, supergelators, hydrogels, drug delivery

## Abstract

Self-assembly of peptide building blocks offers unique opportunities for bottom-up preparation of exquisite nanostructures, nanoarchitectures, and nanostructured bulk materials, namely hydrogels. In this work we describe the synthesis, characterization, gelation, and rheological properties of new dehydrotripeptides, Cbz-*L*-Lys(Cbz)-*L*,*D*-Asp-∆Phe-OH and (2-Naph)-*L*-Lys(2-Naph)-*L*,*D*-Asp-∆Phe-OH, containing a *N*-terminal lysine residue *N_α_*_,*ε*_-*bis*-capped with carboxybenzyl (Cbz) and 2-Naphthylacetyl (2-Naph) aromatic moieties, an aspartic acid residue (Asp), and a *C*-terminal dehydrophenylalanine (∆Phe) residue. The dehydrotripeptides were obtained as diastereomeric mixtures (*L*,*L*,*Z* and *L*,*D*,Z), presumably via aspartimide chemistry. The dehydrotripeptides afforded hydrogels at exceedingly low concentrations (0.1 and 0.04 wt%). The hydrogels revealed exceptional elasticity (G’ = 5.44 × 10^4^ and 3.43 × 10^6^ Pa) and self-healing properties. STEM studies showed that the diastereomers of the Cbz-capped peptide undergo *co-assembly*, generating a fibrillar 3D network, while the diastereomers of the 2-Naph-capped dehydropeptide seem to undergo self-sorting, originating a fibril network with embedded spheroidal nanostructures. The 2-Naph-capped hydrogel displayed full fast recovery following breakup by a mechanical stimulus. Spheroidal nanostructures are absent in the recovered hydrogel, as seen by STEM, suggesting that the mechanical stimulus triggers rearrangement of the spheroidal nanostructures into fibers. Overall, this study demonstrates that diastereomeric mixtures of peptides can be efficacious gelators. Importantly, these results suggest that the structure (size, aromaticity) of the capping group can have a directing effect on the self-assembly (co-assembly vs. self-sorting) of diastereomers. The cytotoxicity of the newly synthesized gelators was evaluated using human keratinocytes (HaCaT cell line). The results indicated that the two gelators exhibited some cytotoxicity, having a small impact on cell viability. In sustained release experiments, the influence of the charge on model drug compounds was assessed in relation to their release rate from the hydrogel matrix. The hydrogels demonstrated sustained release for methyl orange (anionic), while methylene blue (cationic) was retained within the network.

## 1. Introduction

**Figure 1:**
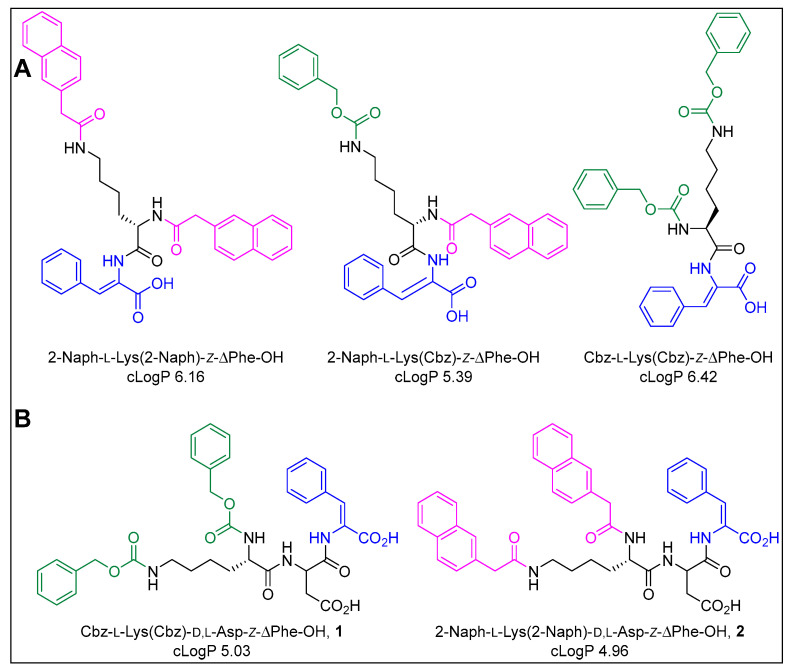
(**A**) Structure of dehydrodipeptide *supergelators* from our previously work [28]. (**B**) Structure of hydrogelators **1** and **2** studied in this work. cLogP values were obtained using https//molinspiration.com (accessed on 29 May 2024).

Tissue and bone engineering, and ultimately regenerative and personalized medicine, seem within reach in a near future thanks to the development of smart functional biomaterials [1,2,3]. Hydrogels are archetypical biomaterials: bulk materials with nanostructuring and high-water content, reminiscent of the extracellular matrix; adjustable rheological properties; biocompatibility and responsiveness to environmental cues [4]. Natural and synthetic, physical (noncovalent) and chemical (cross-linked) polymer-based hydrogels, have emerged during recent decades as effective scaffolds for cell growth and differentiation [5,6,7,8]. Supramolecular (physical) hydrogels based on self-assembly of low molecular weight biological building blocks—peptides, oligosaccharides, and chimeric synthetic molecules (e.g., lipopeptides)—are promising practical and economical alternatives to polymeric hydrogels, owing to their expedited synthesis of building blocks, structural diversity, and structure-tunable rheological properties [9,10,11,12]. The physical nature of self-assembled hydrogels, reliant on an ensemble of weak noncovalent intermolecular forces, electrostatic bonding, van der Walls, hydrophobic, and π–π stacking interactions, warrants intrinsic biocompatibility, degradability, and responsiveness to environmental stimuli [3,13]. Short peptides (di- and tripeptides) *N*-capped with bulky aromatic groups are remarkably effective hydrogelators [9,13]. Environmental stimulation (pH change, heating-cooling cycles, solvent switch, enzymes) triggers molecular self-assembly into a three-dimensional (3D) network of entangled fibers [14,15,16,17,18,19,20]. Trapping of a disproportionate amount of water originates porous hydrogels that allow incorporation, binding, and diffusion of small molecules and cells. Bing Xu and co-workers coined the term π-gelators to emphasize the fundamental contribution of aromatic *N*-capping groups to molecular self-assembly and gelation of low molecular weight peptides [9]. An effective molecular gelator must display high propensity for molecular aggregation and gelation at the lowest possible concentration (critical gelation concentration—*cgc*), thus minimizing potential toxicity and ensuring wide availability and affordability [21,22]. The contribution of our research group to the *peptide soft materials* field is centered around the synthesis of peptide architectures featuring dehydroamino acids as building blocks for preparation of self-assembled hydrogels as drug-delivery and theragnostic platforms [23,24]. Proteolytic resistance is of paramount importance for in vitro and in vivo applications, precluding premature deactivation of active peptide materials. The noncanonical dehydroamino acid residue, in addition to endowing the peptides with proteolytic stability, also adds conformational restrictions to the peptide backbone that favors molecular aggregation and gelation [23]. One of the main hurdles limiting wider use of peptide based self-assembled hydrogels in biomedicine is still their high cost, associated with elaborate long synthetic pathways, often created by solid-phase synthesis. Therefore, reducing the molecular complexity of peptide hydrogelators (minimalist hydrogelators), while retaining high aggregation and gelation propensity, would allow to expedite their synthesis, by simple scalable synthetic pathways [16]. The *cgc* values for di- and tripeptides *N*-capped with bulky aromatic groups are typically in the range 0.1–1.0 wt%. Peptides exhibiting exceptional gelation propensity, *supergelators* and *hypergelators* (*cgc* around 0.01 and 0.001 wt%, respectively), are highly sought [25,26]. Gazit and coworkers described a dipeptide (Fmoc-Lys(Fmoc)-Asp(OH)-OH) *hypergelator* characterized by an extremely low *cgc* (0.002 wt%) [27]. This minimalist *hypergelator*, with two bulky aromatic Fmoc moieties on the *N*-terminal Lys residue, requires a *C*-terminal ionizable Asp residue for balancing the hydrophobicity and solubility of the peptide [27]. As the Fmoc protecting group is labile under alkaline conditions, potentially releasing toxic degradation products, the hydrogels were not accessible by the pH drop methodology, but instead had to be prepared at neutral pH by the solvent switch (DMSO → water) methodology. Deploying stable and more biocompatible Cbz and 2-Naph aromatic protecting groups, our research group disclosed recently a family of minimalist dehydrodipeptide *supergelators* (X)-Lys(Y)-∆Phe-OH (X,Y = Cbz or 2-Naph and X = 2-Naph and Y = Cbz), whose hydrogels could be prepared in aqueous conditions by the pH drop methodology, by addition of *D*-glucono-δ-lactone (Figure 1) [28]. Building on our previous work, herein we describe the synthesis and characterization of novel dehydrotripeptide *supergelators* (X)-Lys(Y)-Asp(OH)-∆Phe-OH (X,Y = Cbz; and X,Y = 2-Naph) (Figure 1) [29]. Interestingly, racemization of the Asp stereogenic center was detected, as signal duplication, in the ^1^H and ^13^C NMR spectra spectroscopy of the dehydrotripeptides. The final dehydrotripeptides and their hydrogels were obtained and studied as equimolar mixtures of diastereomers. Diastereomers are different chemical entities, with different physical-chemical, biological and self-assembly properties. Stereochemistry determining the conformational properties of molecules ends up also governing self-assembly through molecular packing effects. Gazit and co-authors demonstrated that while homochiral peptides L-Phe-L-Phe and D-Phe-D-Phe undergo fast self-assembly in salt-rich aqueous solutions, their heterochiral counterparts L-Phe-D-Phe and D-Phe-L-Phe failed to form ordered structures [30]. Smith and co-workers studied the self-assembly of diastereomeric peptide amphiphiles (C16-L-Ala-L-Lys/C16-D-Ala-D-Lys) and (C16-D-Ala-L-Lys/C16-L-Ala-D-Lys), concluding that the homochiral (LL and DD) diastereomeric pair display comparable but much lower critical micelle concentrations (stronger molecular association) than the heterochiral LD/DL pair [31].

The self-assembly of peptide mixtures, either structurally different peptides or diastereomers, into supramolecular hydrogels, can conceptually follow *co-assembly or self-sorting* pathways. In co-assembly, different peptides combine orderly or randomly to form well-defined fibers (containing both peptides) that further originate the hydrogel 3D network. Otherwise, different peptides can undergo separate self-assembly, *self-sorting*, originating two different well defined fibrous networks. Owing to structural mismatch (molecular packing effects) structurally different peptides are well known to form self-sorted self-assembled hydrogels. Recently, Adams and co-workers studied the self-assembly of diatereomeric mixtures (*L*,*L* and *L*,*D*) of the peptide hydrogelator (2-Naph)-Phe-Phe-OH across a wide range of different molar fractions. These authors demonstrated *self-sorting* of the diastereomers both in the micellar state, at alkaline pH, and in the gel phase, at low pH. Moreover, the authors concluded that the *self-sorting* observed in the micellar state, determined by peptide´s chirality, ends up transferred into the gel state [32].

## 2. Results and Discussion

### 2.1. Synthesis and Gelation Studies

**Scheme 1:**
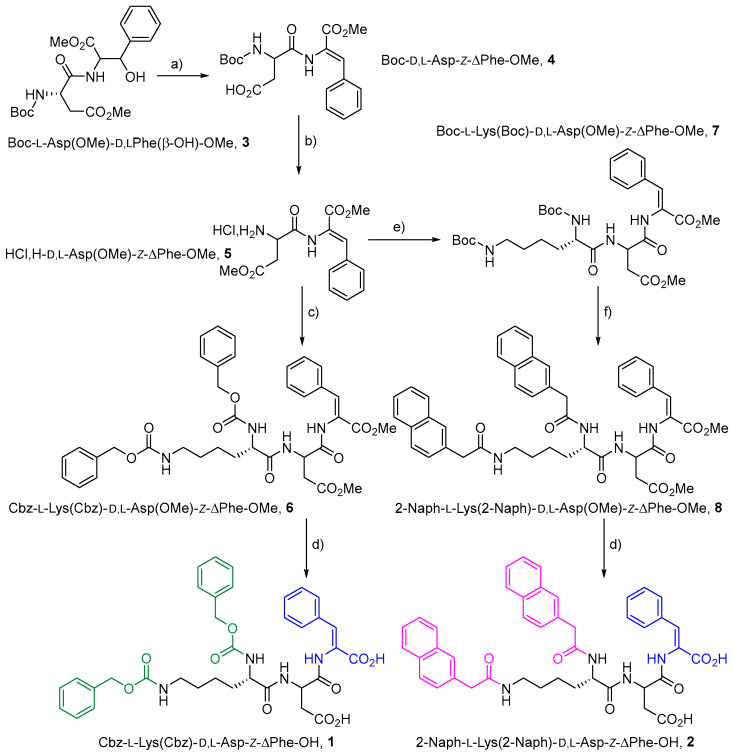
Synthesis of dehydrotripeptides **1** and **2**. (a) (i) Boc_2_O, DMAP, (ii) TMG; (b) SOCl_2_, MeOH. (c) Cbz-Lys(Cbz)-OH, HBTU, Et_3_N; (d) (i) NaOH (1 M), (ii) KHSO_4_ (1 M); (e) Boc-L-Lys(Boc)-OH, HBTU, Et_3_N; (f) (i) TFA, (ii) 2-Naphthylacetic acid, HBTU, Et_3_N.

**Scheme 2:**
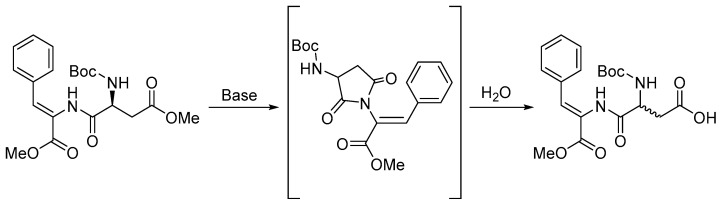
Proposed mechanism for racemization of aspartic acid through aspartimide formation during the dehydration reaction and work-up (adapted from Ref. [35]).

Dehydrotripeptides **1** and **2** were obtained as mixture of diastereomers (*L*,*L*,*Z* and *L*,*D*,*Z*) by conventional solution-phase peptide synthesis using 2-(1H-benzotriazole-1-yl)-1,1,3,3-tetramethylaminium hexafluorophosphate (HBTU) as coupling reagent (Scheme 1). The dehydration step involved treatment of dipeptide **3** with di-*tert*-butyl dicarbonate (Boc_2_O) and 4-dimethylaminopyridine (DMAP), followed by *N*,*N*,*N’*,*N’*-tetramethylguanidine (TMG) [33]. In this reaction, the methyl ester group in the aspartic acid side chain was removed, presumably through aspartimide formation [34,35]. The aspartimide was spontaneously generated during the dehydration reaction, presumably by nucleophilic attack of the amide nitrogen of the dehydroamino acid on the β-carboxylic acid group of aspartic acid (Scheme 2). Compound **4** was reacted with thionyl chloride in methanol to generate the *N*-deprotected-*C*,*C*-diprotected dehydrodipeptide **5.** Hydrogelator **1** was directly obtained from compound **5** by coupling with Cbz-*N_α_*_,*ε*_-*bis*-protected Lys (Cbz-L-Lys(Cbz)-OH), followed by saponification. Hydrogelator **2** was synthesized from compound **5** by a three-step procedure: coupling with Boc-*N_α_*_,*ε*_-*bis*-protected Lys (Boc-L-Lys(Boc)-OH), Boc deprotection and coupling with 2-Naphthylacetic acid, followed by saponification (Scheme 1). Racemization is likely traced to the dehydration reaction and ensuing aspartimide formation, given the high propensity of aspartimides towards racemization (Scheme 2). Racemization could only be first identified, by signal duplication in the ^1^H and ^13^C NMR spectra, in the protected dehydrotripeptides **6** and **8** and afterwards in the final compounds **1** and **2** (*L*,*L*,*Z* and *L*,*D*,*Z*) (Scheme 1 and Scheme 2). No attempt was made to separate the diastereomers of hydrogelators **1** and **2**. Hydrogels **1** and **2** were obtained using the diastereomeric mixtures.

**Figure 2:**
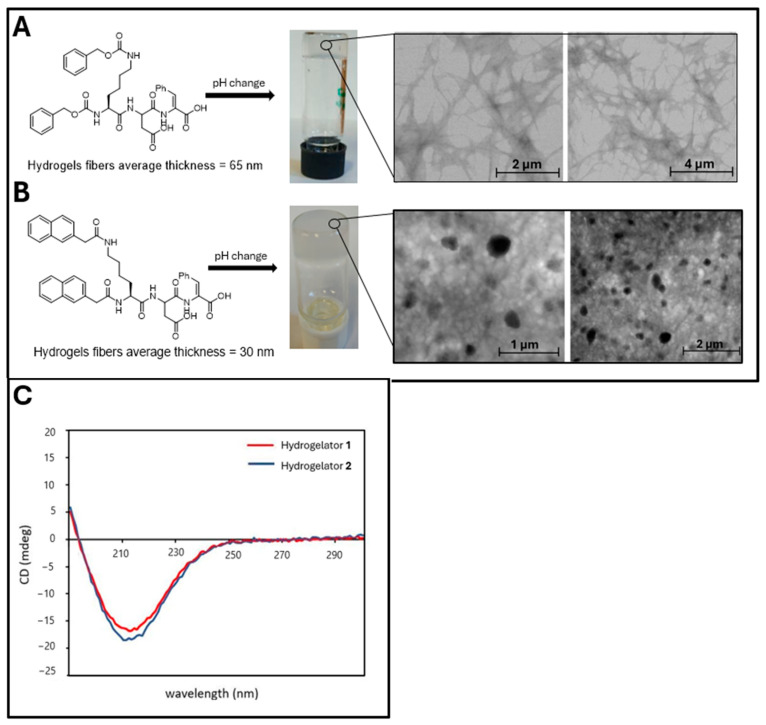
(**A**) STEM images of hydrogel of **1**. (**B**) STEM images of hydrogel of **2**. (**C**) CD spectra of aqueous solutions of hydrogelators **1** and **2** (0.01 wt%).

Hydrogels **1** and **2** were prepared by the *D*-glucono-δ-lactone (GDL) pH dropping methodology. Initially, peptide dispersions were made water soluble by pH adjustment to around 10, by addition of NaOH 1M under vigorous magnetic stirring. Next, gradual hydrolysis of added GDL to gluconic acid was used to trigger gelation by pH drop. As the hydrolysis rate of GDL is slower than the diffusion rate of its acidic product, homogeneous hydrogels were obtained by the GDL methodology [8]. Dehydrotripeptides **1** and **2** (as diastereomeric mixtures) produced free-standing hydrogels by the GDL pH dropping methodology. The *critical gelation concentration* (CGC) was determined by tube inversion using a wide range of peptide concentrations (Table 1, Figure 2A,B).

The CGC values determined for dehydrotripeptides **1** and **2** (as diastereomer mixtures) (0.1 and 0.04 wt%, respectively) are remarkably low comparing to those reported for mono *N*-capped dehydropeptides reported by the research group, generally around 0.4 wt% [24]. Interestingly, dehydrotripeptides **1** and **2** display CGC values of the same order of magnitude as those reported for analogous dehydrodipeptides Cbz-L-Lys(Cbz)-*Z*-ΔPhe-OH and (2-Naph)-L-Lys(2-Naph)-*Z*-ΔPhe-OH (0.2 and 0.05 wt%, respectively) (Figure 1 and Table 1) [28]. Therefore, introduction of the (polar) aspartic acid residue does not compromises the self-assembly propensity and gelation ability of the dehydrotripeptides (Table 1) [28]. Dehydrotripeptides **1** and **2** display much higher CGC (0.1 and 0.04 wt%) than the *hypergelator* Fmoc-L-Lys(Fmoc)-L-Asp-OH (CGC 0.001 wt%), despite exhibiting similar *lipophilicity*; cLogP values for Fmoc-L-Lys(Fmoc)-L-Asp(OH)-OH and for dehydrotripeptides **1** and **2** are of the same order of magnitude (5.51, 5.03, and 4.96, respectively) [27]. These results probably reflect the contribution of stronger intermolecular π–π stacking interactions, provided by the larger *Fmoc* group, to the self-assembly process. Importantly, the (polar) Asp residue contributes to make the dehydrotripeptides soluble in alkaline solution which allows GDL gelation. Recently, the hydrogelation of diastereomeric mixtures (*L*,*L*) and (*L*,*D*) of the dipeptide [2-(naphthalen-2-yloxy)acetyl]phenylalanylphenylalanine ((2-Naph)-Phe-Phe(OH)) was studied at a concentration of 0.02 mM (1.0 wt%), considerably higher than the CGC displayed the diastereomeric mixtures (L,L,Z and L,D,Z) of dehydrotripeptides **1** and **2** [32].

### 2.2. Scanning Transmission Electron Microscopy (STEM)

The micro-nanostructuring of hydrogels **1** and **2** was studied by STEM. Hydrogel **1** displays entangled fibers with average thickness 65 nm (Figure 2A). Hydrogel 2 shows a fibril network with embedded spheroidal nanostructures (Figure 2B). Presumably, the diastereomers of dehydropeptide **2** undergo independent (separate) self-assembly originating a *self-sorted* hydrogel. The STEM images of hydrogel **1** suggest co-assembly of the diastereomers. Cryo-TEM images of a *self-sorted* hydrogel obtained from a diastereomeric mixture (*L*,*L* and *L*,*D*) of peptide hydrogelator (2-Naph)-Phe-Phe(OH) show coexistence of large nanotubes and thinner structures [32]. The low resolution of the STEM images of hydrogel **1** does not exclude the possibility of self-sorted assembly of the diastereomers into fibers with different aspect ratios. The difference in self-assembly behavior observed for dehydrotripeptides **1** and **2**, *co-assembly* vs. *self-sorting* of diastereomers, respectively, must be ascribed to the aromatic caping moiety. The 2-Naph group, bulkier and stereochemically more demanding, is likely to induce a conformation mismatch that disfavors the co-assembly of the diastereomers of dehydropeptide **2**. Therefore, the self-assembly fate, *co-assembly* or *self-sorting*, of a diastereomeric mixture of peptides seems to be controlled by the properties of the aromatic capping group.

### 2.3. Circular Dichroism (CD) Studies

The circular dichroism (CD) spectra of *supergelators* **1** and **2** are shown in Figure 2C. These spectra were acquired using dehydrotripeptide solutions at concentrations well below the CGC to minimize light scattering effects, common in turbid hydrogels. During sample preparation, D-glucono-δ-lactone (GDL) was added to alkaline solutions of dehydrotripeptides **1** and **2** to replicate the hydrogel formation conditions. The CD spectra for hydrogelators **1** and **2** exhibit strong similarity, both displaying a negative band at 215 nm, indicative of a dominant β-sheet structure (Figure 2C). These spectra closely resemble those previously reported for dehydrodipeptide *supergelators* [28].

### 2.4. Rheological Studies

**Figure 3:**
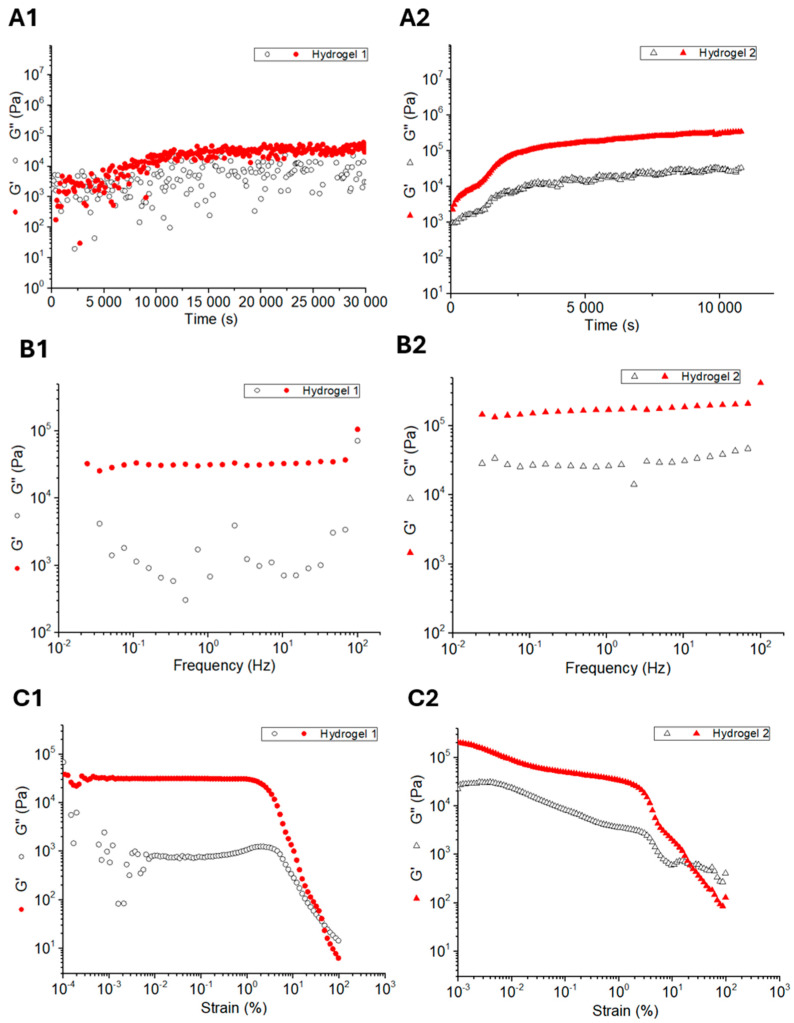
(**A**) Elastic (G’) and viscous (G’’) moduli during the kinetics process of hydrogelation of **1** (**A1**) and **2** (**A2**) at 0.2 wt%. (**B**) Frequency dependence of the shear elastic G’ and G’’ moduli of **1** (**B1**) and **2** (**B2**) at 0.2 wt%. (**C**) Strain dependence of the shear elastic G’ and loss G’’ moduli of **1** (**C1**) and **2** (**C2**) at 0.2 wt%.

Rheological studies offer insights into the structural properties of gels, including the type, number, and stiffness of the fibrillar network responsible for gelation. The gelation kinetics of dehydrotripeptides **1** and **2** reveals that hydrogel **2** attains a G’ (storage/elastic modulus) value significantly higher than G’’ (loss/viscosity modulus) within 2.8 h, indicating rapid gelation, comparable to that observed for ultrashort *N*-capped dehydropeptides [24,28]. In contrast, hydrogelator **1** exhibits slower gelation kinetics, requiring over 4 h to reach the maximum G’ value. Hydrogel **2** seems suitable for applications that require in vivo injection and in situ gelation. When structural equilibrium was reached, with stable maximum G’ and G’’ values over time, the mechanical spectrum of gels **1** and **2** was determined by performing a frequency sweep, from 100–0.1 Hz, while maintaining a constant strain (0.001%) (Figure 3B). For both hydrogels **1** and **2**, G’ remains essentially constant across the tested frequency range, while G’’ for hydrogel **1** shows a local minimum. As expected, G’ exceeds G’’ for both hydrogels (Table 2).

Hydrogel **2** (0.2 wt%, GDL) displays significantly higher elasticity (circa 100×) than hydrogel **1** and the homochiral hydrogel (*L*,*L*)-(Fmoc-Lys(Fmoc)-Asp-OH (G’= 1 × 10^4^ Pa, 0.5 wt%, solvent switch DMSO→H_2_O) described as *hypergelator* by Gazit and coworkers [27]. Dehydrotripeptides **1,** and especially **2,** as diastereomeric mixtures are very efficacious hydrogelators. After the frequency sweep, hydrogels **1** and **2** were submitted to a strain sweep, where the frequency was fixed at 1 Hz (Figure 3C). Hydrogel **2** breaks up more easily than hydrogel **1**, at a strain of 21.5% and 55.5%, respectively. Although more elastic, hydrogel **2** breaks up more easily than hydrogel **1,** suggesting that the embedded spheroidal nanostructures weaken the fibrillar network of hydrogel **2** (Figure 2B and Figure 3C).

**Figure 4:**
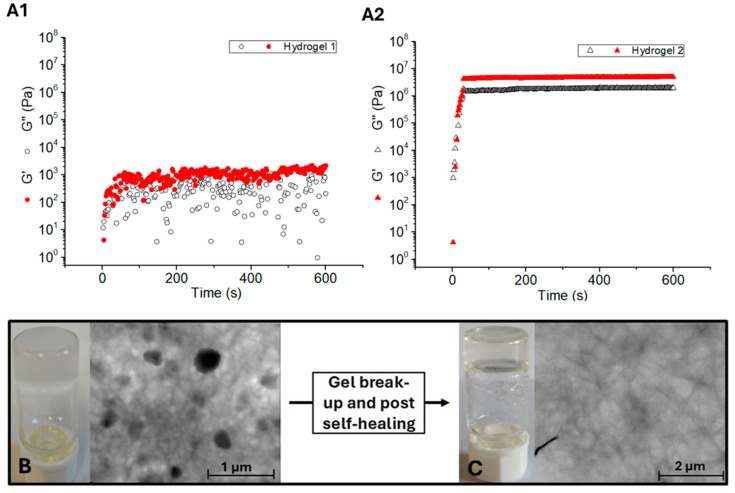
(**A**) Elastic and viscous modulus during the second kinetic process of hydrogels **1** (**A1**) and **2** (**A2**) at 0.2 wt%. (**B**) Optical and STEM images of hydrogel formed by **2**. (**C**) Optical and STEM images of hydrogel **2** after second gelification.

After breaking up, a second kinetics was acquired for hydrogels **1** and **2**, implying self-healing properties (Figure 4A). Hydrogel **1** reforms within one minute after break up, although with incomplete recovery of the rheological properties, G’ value of 1.38 × 10^3^ Pa and a G’’ value of 505 Pa. In contrast, hydrogel **2** reformed also within 1 min with complete recovery of the rheological properties, G’ value of 4.91 × 10^6^ Pa and a G’’ value of 1.93 × 10^6^ Pa. Interestingly, hydrogel **2** before breaking up is turbid while the recovered hydrogel is transparent. The corresponding STEM images reveal that spheroidal nanostructures are absent in the recovered hydrogel (Figure 4B,C), indicating that the mechanical stimulus triggers rearrangement of the spheroidal nanostructures into fibers.

### 2.5. Biocompatibility and Cytotoxicity Studies

**Figure 5:**
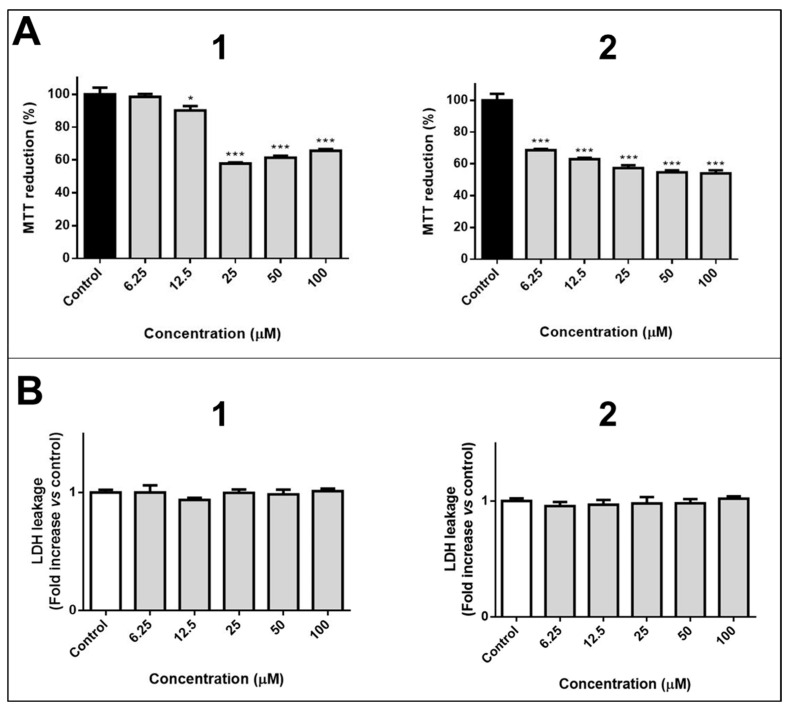
Concentration dependence of hydrogelators **1** and **2** on: (**A**) Viability of HaCaT cells over 24 h; (**B**) LDH activity in the culture media of HaCaT cells over 24 h. (* *p* < 0.05, *** *p* < 0.001); Triton X-100 was used as a positive control to lyse cells.

Initial evaluation of dehydropeptides **1** and **2**, regarding potential effects on cell viability, was performed with human keratinocytes, HaCaT cell line. Hydrogelator **1** showed a significant impact on cell viability at concentrations above 12.5 µM, causing an apparent loss of cell viability of around 40%. Further increase in concentration, up to 100 µM, seems not to result in further loss of cell viability. Hydrogelator **2** elicited also an apparent loss of cell viability around 40% already at the lowest tested concentration (6.25 µM) and a very low decrease of cell viability with further increase in concentration, up to 100 µM (Figure 5A). The low concentration dependence of the toxicity observed for both dehydropeptide **1** and **2** suggests a physical mechanism of toxicity, i.e., not dependent on the interaction between the peptides and a molecular target.

The lactate dehydrogenase (LDH) leakage assay evaluates the extent of membrane disruption elicited by test compounds. Increased leakage of cytosolic LDH is a hallmark of necrosis. Dehydropeptides **1** and **2** in concentrations up to 100 μM did not trigger any significant increase in LDH leakage by HaCaT cells, excluding loss of membrane integrity and necrosis as the mechanism leading to loss of cell viability.

### 2.6. Drug Delivery Studies

**Figure 6:**
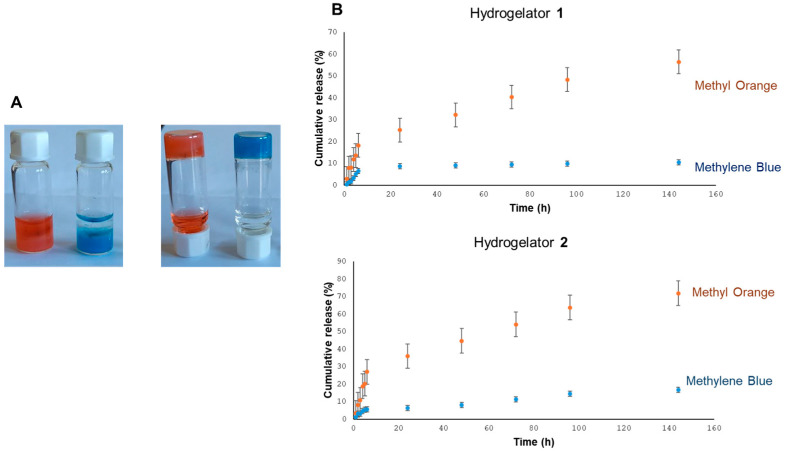
(**A**) Representative images of hydrogels **1** and **2** loaded with methyl orange (left), and methylene blue (right) layered with PBS solution (1.5 mL) after 168 h. (**B**) Cumulative release of methylene blue and methyl orange from hydrogels **1** and **2** over time.

Supramolecular hydrogels show great potential as drug delivery systems, alleviating pharmacokinetics limitations experienced by some drugs, related to reduced aqueous solubility and short half-lives in vivo. Hydrogels **1** and **2** were prepared and investigated for their capacity to incorporate and release cationic and anionic dyes (methylene blue (MB) and methyl orange (MO), respectively) as drug models (Figure 6A). Dye-loaded hydrogels **1** and **2** were prepared by the GDL methodology (Section 2.1) replacing water by dye solutions [36]. The cumulative release of dyes, into a layer of water on the top the hydrogel surface, was monitored overtime by UV-Vis spectroscopy. Visual inspection showed that a very small amount of MB was released from the hydrogel´s matrix after 7 days (Figure 6A). UV-Vis spectroscopy revealed that only 10% and 16% of the loaded cationic MB dye was released after 7 days from hydrogels **1** and **2**, respectively (Figure 6B). Hydrogels **1** and **2** released the anionic dye MO in much higher extent (56% and 71%, respectively) after 7 days (Figure 6B). These data indicate that the dyes interact electrostatically with the hydrogel’s fibers, presumably anionic, owing to ionization of the *C*-terminal of the peptide hydrogelators.

**Figure 7:**
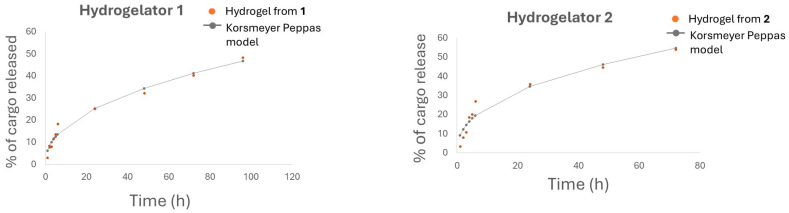
Korsmeyer–Peppas model applied to the release of MO from hydrogelators **1** and **2**.

Various mathematical models were used to evaluate quantitatively the drug release from hydrogels **1** and **2**. The Korsmeyer–Peppas model, which accounts for both diffusion and erosion, provided the best fit to the data (Figure 7) [37]. The fitting parameters determined for this model show faster release (higher *k* value) of methyl orange from hydrogel **2** than from hydrogel **1**. MO release by both hydrogels is governed by a diffusion-controlled mechanism (*n* value < 0.5) (Table 3 and Equation (1)).

(1) $$\frac{M_{t}}{M}={kt}^{n}$$

$M_{t}$ = amount of cargo released at time t;

M = total amount of cargo used for the release study;

k = release rate constant incorporating structural and geometric characteristics of drug dosage form;

n = release exponent;

Equation (1), Korsmeyer–Peppas model. Adapted from Ref. [37].

**Table 3:** Release coefficients of the Korsmeyer–Peppas model obtained for methyl orange release profiles of hydrogels **1** and **2**.

Hydrogelator	*k*	*n*	*R* ^2^
**1**	6.1203	0.4459	0.9892
**2**	9.2804	0.4147	0.9713

## 3. Conclusions

In this study we disclose the synthesis, characterization, and gelation properties of new dehydrotripeptide *supergelators* containing a *N*-terminal lysine residue *bis*-*N_α_*_,*ε*_-capped with Cbz and 2-Naph aromatic moieties, a (racemic) aspartic acid residue and a *C*-terminal dehydrophenylalanine residue. The synthetic pathway afforded the dehydrotripeptide hydrogelators as diastereomeric mixtures, presumably via aspartimide chemistry. Studied as diastereomeric mixtures, the dehydropeptides revealed exceptional (*super*)gelators *(cgc* 0.1 and 0.04 wt%, for dehydrotripeptides **1** and **2**, respectively) with their hydrogels displaying exceptional elasticity (G´= 5.44 × 10^4^ and 3.43 × 10^6^ Pa for hydrogel **1** and **2**, respectively). STEM studies suggests that the diastereomers of dehydropeptide **1** undergo co-assembly, generating a fibrillar 3D network, while the diastereomers of dehydropeptide **2** seemingly undergo self-sorting, originating a fibril network with embedded spheroidal nanostructures. Both hydrogels display fast recovery after mechanical breakup, with hydrogel **2** showing complete recovery. Spheroidal nanostructures are absent in recovered hydrogel **2** suggesting that the mechanical stimulus triggers rearrangement of the spheroidal nanostructures into fibers. The toxicity low concentration dependence observed for both dehydropeptides suggests a physical mechanism of toxicity, i.e., not dependent on the molecular interaction between the peptides and a molecular target. Loss of membrane integrity (necrosis) can be excluded as the toxicity mechanism. Hydrogels **1** and **2** revealed effective nanocarriers for sustained delivery of dyes, as drug model compounds, through a diffusion-controlled release mechanism.

Overall, this study shows that diastereomeric mixtures of peptides are very efficacious gelators. Importantly, our results suggest that the properties (size, aromaticity) of the capping group can have a directing effect on the self-assembly fate—*co-assembly* vs. *self-sorting*—of diastereomers.

## 4. Materials and Methods

### 4.1. Synthesis

Gelators **1** and **2** were synthesized using procedures developed by our research group. Compounds were characterized by ^1^H and ^13^C NMR spectroscopy and by High-Resolution Mass Spectrometry (HRMS). Detailed experimental procedures and characterization data for compounds **3**–**11** can be found as Supplementary Material. NMR spectra were acquired by a Bruker Avance III 400 spectrometer (Bruker, Billerica, MA, USA) operating at 400.13 MHz for ^1^H and 100.62 MHz for ^13^C. HRMS data were provided by the mass spectrometry service at the University of Vigo, Spain. The partition coefficient (log *p*) between water and *n*-octanol was calculated for gelators **1** and **2** using Molinspiration Cheminformatics software (Molinspiration, Slovensky Grob, Slovak Republic, 2017; https://www.molinspiration.com, accessed on 9 May 2022). The log *p* value, calculated as the sum of fragment-based contributions and correction factors, serves as a quantitative measure of the compounds’ lipophilicity.

#### 4.1.1. Synthesis of Cbz-L-Lys(Cbz)-D,L-Asp(OH)-Z-ΔPhe-OH, 1

Cbz-L-Lys(Cbz)-D,L-Asp(OMe)-*Z*-ΔPhe-OMe was dissolved in 1,4-dioxane (27.5 mL) and a solution of 1.0 M NaOH (3.0 equiv, 16 mL, 0.824 mmol) was added. The reaction was followed by TLC until no starting material was detected (typically about 4 h). The organic solvent was removed under reduced pressure, and the reaction mixture was acidified to pH 2–3 with KHSO_4_ (1 M). The solid was collected by filtration, then washed with Et_2_O. The solid was identified as a 1:1 diastereomeric mixture of Cbz-L-Lys(Cbz)-D,L-Asp(OH)-*Z*-ΔPhe-OH, **1** (0.196 g, 50%).

^1^H NMR (400 MHz, DMSO-*d*_6_) δ: 1.13–1.41 (4H, m, γ-C*H*_2_ and δ-C*H*_2_ of Lys), 1.48–1.69 (2H, m, β-C*H*_2_ of Lys), 2.59–2.71 (1H, m, β-C*H*_A_H_B_ of Asp), 2.78–2.87 (1H, m, β-CH_A_C*H*_B_ of Asp), 2.88–2.97 (2H, m, ε-C*H*_2_ of Lys), 3.90–4.02 (1H, m, α-C*H* of Lys), 4.51–4.65 (1H, m, α-C*H* of Asp), 4.99 (2H, s, 1 × C*H*_2_ of Cbz), 5.03 (2H, s, 1 × C*H*_2_ of Cbz), 7.10–7.42 (16H, Ar*H* and β-*H* of ΔPhe and 2 × N*H*), 7.59–7.71 (2H, m, Ar*H*), 8.20 (1H, d, J 8.0 Hz, N*H* of Asp), (9.57 and 9.73 (1H, s, N*H* of ΔPhe)), 12.45 (2H, s, CO_2_*H* of Asp and CO_2_*H* of ΔPhe).

^13^C NMR (100.6 MHz, DMSO-*d*_6_, δ): 22.7 (CH_2_, γ-*C*H_2_ of Lys), 29.1 (CH_2_, δ-*C*H_2_ of Lys), 31.6 (CH_2_, β-*C*H_2_ of Lys), 36.9 (CH_2_, β-*C*H_2_ of Asp), 40.1 (CH_2_, ε-C*H*_2_ of Lys), (48.3 and 48.5 (CH, α-*C*H of Asp)), 54.5 (CH, α-*C*H of Lys), 65.1 (CH_2_, 1 × *C*H_2_ of Cbz), 65.4 (CH_2_, 1 × *C*H_2_ of Cbz), 127.70 (CH, Ar), 127.76 (CH, Ar), 128.3 (CH, Ar), 128.5 (CH, Ar), 128.6 (CH, Ar), 129.1 (CH, Ar), 129.4 (CH, Ar), 130.01 (CH, Ar), 130.08 (CH, Ar), 131.2 (CH, β-*C*H of ΔPhe), 133.6 (C, α-*C* of ΔPhe), 137.0 (C, Ar), 137.3 (C, Ar), 165.4 (C, *C*=O), 166.3 (C, *C*=O), 169.2 (C, *C*=O), 169.5 (C, *C*=O), 171.8 (C, *C*=O); 172.5 (C, *C*=O). HRMS (ESI) *m/z*: [M + H]^+^ calcd for C_35_H_39_N_4_O_10_ 675,26607; found 675,26494.

#### 4.1.2. Synthesis of Naph-L-Lys(Naph)-D,L-Asp(OH)-Z-ΔPhe-OH, 2

Naph-L-Lys(Naph)-D,L-Asp(OMe)-Z-ΔPhe-OMe was dissolved in 1,4-dioxane (27.5 mL) and a solution of 1 M NaOH (3.0 equiv, 16 mL, 0.824 mmol) was added. The reaction was followed by TLC until no starting material was detected (typically about 4 h). The organic solvent was removed under reduced pressure, and the reaction mixture was acidified to pH 2–3 with KHSO_4_ (1 M). The solid was collected by filtration, then washed with Et_2_O. The solid was identified as a 1:1 diastereomeric mixture of Naph-L-Lys(Naph)-D,L-Asp(OH)-Z-ΔPhe-OH, **2** (0.200 g, 98%).

^1^H NMR (400 MHz, DMSO-*d*_6_) δ: 1.16–1.41 (4H, m, γ-C*H*_2_ and δ-C*H*_2_ of Lys), 1.43–1.71 (2H, m, β-C*H*_2_ of Lys), 2.53–2.62 (1H, m, β-C*H*_A_H_B_ of Asp), 2.66–2.75 (1H, m, β-CH_A_C*H*_B_ of Asp), 2.86–3.07 (2H, m, ε-C*H*_2_ of Lys), 3.53–3.68 (4H, m, 2 × Naph C*H*_2_), 4.22–4.32 (1H, m, α-C*H* of Lys), 4.53–4.62 (1H, m, α-C*H* of Asp), 7.15–7.50 (10H, m, Ar*H* and β-C*H* of ΔPhe), 7.53–7.90 (10H, m, Ar*H* and 2 × N*H*), 7.99–8.10 (1H, m, Ar*H*), 8.22–8.37 (2H, m, 2 × N*H*), (9.57 and 9.62 (1H, s, N*H* of ΔPhe)).

^13^C NMR (100.6 MHz, DMSO-d_6_, δ): 22.7 (CH_2,_ γ-*C*H_2_ of Lys), 28.8 (CH_2_, δ-*C*H_2_ of Lys), 32.0 (CH_2_, β-*C*H_2_ of Lys), 36.9 (CH_2_, β-*C*H_2_ of Asp), 38.6 (CH_2_, ε-*C*H_2_ of Lys), 42.2 (CH_2_, 1 × *C*H_2_ of Naph), 42.5 (CH_2_, 1 × *C*H_2_ of Naph), (48.5 and 49.7 (CH, α-*C*H of Asp)), 52.3 (CH, α-*C*H of Lys), 125.5 (CH, Ar), 126.10 (CH, Ar), 126.14 (CH, Ar), 127.2 (CH, Ar), 127.4 (CH, Ar), 127.5 (CH, Ar); 127.62 (CH, Ar), 127.67 (CH, Ar), 127.7 (CH, Ar), 128.5 (CH, Ar), 128.62 (CH, Ar), 128.63 (CH, Ar), 129.2 (CH, Ar), 130.01 (CH, Ar), 130.06 (CH, Ar), 130.1 (CH, Ar), 130.2 (CH, Ar), 131.3 (CH, β-*C*H of ΔPhe), 131.8 (C, Ar), 133.04 (C, Ar), 133.05 (C, Ar), 133.5 (C, α-*C* of ΔPhe), 133.6 (C, Ar), 134.15 (C, Ar), 134.16 (C, Ar), 134.2 (C, Ar), (166.3 and 166.4 (C, *C*=O)), (169.2 and 169.3 (C, *C*=O)), (170.01 and 170.03 (C, *C*=O)), (170.11 and 170.13 (C, *C*=O)), (171.64 and 171.67 (C, *C*=O)), 172.5 (C, *C*=O). HRMS (ESI) *m/z*: [M + H]^+^ calcd for C_43_H_43_N_4_O_8_ 743,30734; found 743,30727.

### 4.2. Preparation of Hydrogels and Determination of the Critical Gelation Concentration (cgc)

Dispersions of hydrogelators **1** and **2** (0.3, 0.4, 0.5, 0.6, 0.7, 0.8, 0.9, 1.0, 2.0, and 3.0 mg) in water (1 mL) were made soluble by addition of small aliquots (circa 30 µL) of aqueous NaOH (1 M) under vigorous magnetic stirring until pH 10. Further sonication, for approximately 1 min, afforded solutions of gelators **1** and **2** in the concentration range 0.03–0.3 wt%. Next, *D*-glucono-δ-lactone (GDL) (4 mg) was added to each vial followed by thorough mixing for 1 min. The solutions were left undisturbed overnight at room temperature. Gel formation was identified by the tube inversion test, i.e., tubes showing free-standing material after inversion for 5 min were classified as gels. The minimal concentration of hydrogelators **1** and **2** required for gelation (CMC) was 0.1 and 0.04 wt%, respectively.

### 4.3. Scanning Transmission Electron Microscopy (STEM)

Scanning transmission electron microscopy (STEM) images were acquired using a NanoSEM-FEI Nova 200 (FEI Technologies, Inc., Hillsboro, OR, USA) operating at 15 kV. The system is equipped with an Energy Dispersive Spectroscopy (EDS) analyzer and an Electron Backscatter Diffraction (EBSD) detection system, specifically the EDAX—Pegasus X4M (SEMAT (Material Characterization Services), Guimarães, Portugal). Small portions of hydrogels **1** and **2** were placed onto TEM 400 mesh copper grids coated with Formvar/Carbon. Excess material was removed. ImageJ 1.53 software (National Institutes of Health (NIH), Bethesda, MD, USA) was used for processing the STEM images regarding enhancement of local contrast local and brightness adjustment, and manual selection of fibers.

### 4.4. Circular Dichroism

Circular dichroism CD spectra were recorded using a Jasco spectropolarimeter, model J-1500 (JASCO, Tokyo, Japan), under nitrogen flow, at 25 °C, in 0.1 mm quartz cells using hydrogelator solutions at 0.01 wt%.

### 4.5. Rheological Studies

The viscoelastic characterization of hydrogels was performed at 25 °C in a MCR300 stress-controlled rotational rheometer (Anton Paar GmbH, Graz, Austria) using the Couette cell geometry (1 mL volume and 0.5 mm gap). After loading the gel-forming hydrogelator solutions into the Couette cell, a shear rate of 5 s^−1^ was applied for 1 min to the stress cell, to attain sample homogenization. Gel formation kinetics was acquired over 10 h, by applying a small amplitude oscillatory shear (SAOS), with a frequency of 1 Hz and an amplitude of 0.01%, recording the shear storage (G’) and loss (G”) moduli every 100 s. Mechanical spectra were acquired for the hydrogels by performing a frequency sweep (from 100 to 0.01 Hz) while applying a constant SAOS amplitude (0.01%). Next, to test for gel break-up, a dynamic strain sweep, from 0.0001–100%, was performed at 1 Hz.

### 4.6. Sustained Release Assays

Hydrogels **1** and **2** (1 mL; 0.2 wt%) loaded with methylene blue (0.1 mM) or methyl orange (0.2 mM) were prepared as described above [36]. Water (1 mL) was carefully layered on the top of the hydrogels. Aliquots (100 μL) of the layered solution were removed at set time points and replaced by an equivalent volume of water, to keep the sink conditions. The concentration of released dyes was calculated by UV spectrophotometry in a microplate reader at 465 and 666 nm, for methyl orange and methylene blue, respectively, resorting to standard calibration curves. Cumulative drug release was expressed as mean percentage of triplicate experiments.

### 4.7. Cell Culture

Human keratinocytes, ATCC cell line HaCaT (Manassas, VA, USA) were cultured in DMEM, supplemented with 10% FBS and 1% penicillin/streptomycin, and were incubated at 37 °C in a humidified atmosphere with 5% CO_2_.

### 4.8. MTT Assay

HaCaT cells were seeded in 96-well plates (1.5 × 10^4^ cells/well) and left to attach for 24 h. After, cells were incubated with different concentrations of the test hydrogelators for another 24 h period. Next, cell viability was assessed by the ability of metabolically active cells to reduced MTT to formazan over the course of 2 h. The absorbance of DMSO dissolved formazan crystals was measured at 570 nm in a Multiskan GO plate reader (Thermo Fisher Scientific; Waltham, MA, USA). Cell viability was expressed as mean percentage ± standard error of the negative control (DMSO, 100% viability) of at least three independent experiments performed in triplicate.

### 4.9. LDH Leakage

Cell lysis, elicited by test compounds, and associated to the cell death mechanism necrosis, was evaluated by release of the stable cytosolic enzyme lactate dehydrogenase (LDH) into the assay medium. The release of LDH was determined using a CytoTox 96^®^ assay kit (Promega; Madison, WI, USA) according to the manufacturer’s instructions.

Briefly, cells were seeded in 96-well plates (1.5 × 10^4^ cells per well) and left to attach for 24 h. After, cells were incubated with different concentrations of the test hydrogelators for another 24 h period. Triton X-100 (1%) was used as positive control (100% cell lysis, over 30 min incubation). Culture media aliquots of the assays (40 µL) were transferred to a 96-well plate and the absorbance was measured at 490 nm in a Multiskan GO plate reader (Thermo Fisher Scientific; Waltham, MA, USA). The results are expressed as fold-increase of absorbance in treated vs. untreated cells for three independent experiments performed in duplicate. Following assessment of the distribution of the results, ANOVA was performed using GraphPad Prism 8.0 (GraphPad Prism Inc., San Diego, CA, USA).

## Figures and Tables

**Table 1:** Optimized conditions for gelation of dehydrotripeptides **1** and **2**.

Hydrogelator	Critical Gelation Concentration (CGC) ^a^		pH
	wt%	mM	
Cbz-L-Lys(Cbz)-*Z*-ΔPhe-OH ^b^	0.2	3.57	5.0
2-Naph-L-Lys(2-Naph)-*Z*-ΔPhe-OH ^b^	0.05	0.80	4.8
Cbz-L-Lys(Cbz)-D,L-Asp-*Z*-ΔPhe-OH, 1 ^c^	0.1	0.002	5.2
2-Naph-L-Lys(2-Naph)-D,L-Asp-*Z*-ΔPhe-OH, 2 ^c^	0.04	0.0006	4.8

^a^ GdL concentration used for gelation dehydrotripeptides **1** and **2** was 0.4 wt%. ^b^ From reference [28]. ^c^ This work.

**Table 2:** G’ and G’’ for hydrogels formed from **1** and **2**.

Hydrogel	G’ (Pa)	G’’ (Pa)
**1**	5.44 × 10^4^	7.94 × 10^3^
**2**	3.43 × 10^6^	2.49 × 10^4^

## Data Availability

The original contributions presented in the study are included in the article/supplementary material, further inquiries can be directed to the corresponding authors.

## Supplementary Materials

The following supporting information can be downloaded at: https://www.mdpi.com/article/10.3390/gels10100629/s1. File S1: Experimental procedures and characterization data for compounds **3**, **4**, **5**, **6**, **7**, and **8**.
