# Supplementary material for: New Supramolecular Hydrogels Based on Diastereomeric Dehydrotripeptide Mixtures for Potential Drug Delivery Applications
Source: Gels. 2024 Sep 30;10(10):629. doi: 10.3390/gels10100629 (PMC11507963; doi:10.3390/gels10100629)
Supplement: Supplementary file 1 [file gels-10-00629-s001.zip › gels-3162670-supplementary.pdf]

# Supporting Information

## New Supramolecular Hydrogels Based on Diastereomeric De-hydrotripeptide Mixtures for Potential Drug Delivery Applications

Carlos B.P. Oliveira[1], André Carvalho[1], Renato B. Pereira[2], David M. Pereira[2], Loic Hilliou[3], Peter J. Jervis[1], José A. Martins[1,*] and Paula M.T. Ferreira[1,*]

<u>Experimental procedures and characterisation data for compounds **3**, **4**, **5**, **6**, **7**, and **8.**</u>

Synthesis of Boc-L-Asp(OMe)-D,L-Phe(β-OH)-OMe (**3**):

Boc-L-Asp(OMe)-OH (0.532 g, 2.15 mmol) was dissolved MeCN (5 mL) and cooled to 0 °C. HBTU (1.1 equiv, 0.900 g, 2.37 mmol), (H-Phe-D,L-β-OH)-OMe 1.00 equiv, 0.500 g, 2.15 mmol) and triethylamine (3 equiv, 0.9 mL, 6.47 mmol) were added sequentially, with 2 min between each addition, and then the mixture was stirred at rt overnight. The solvent was removed under reduced pressure to afford a residue that was partitioned between EtOAc (50 mL) and $KHSO_4$ (50 mL, 1 M). After separation of the phases, the organic layer was thoroughly washed with $KHSO_4$ (1 M, 2 x 50 mL), $NaHCO_3$ (1 M, 2 x 50 mL) and brine (2 x 50 mL) and then dried with $MgSO_4$. Filtration followed by removal of the solvent under reduced pressure afforded a diastereomeric mixture of Boc-L-Asp(Me)-D,L-Phe(β-OH)-OMe as a white solid (0.425 g, 47%). $^1$H NMR (400 MHz, DMSO-$d_6$) δ: 1.46 and 1.47 (9H, 2 s, OC(C$H_3$)$_3$, 2.48-2.49 and 2.51-2.57 (2H, d, J 6.0 Hz, β-C$H_2$ of Asp), 2.69-2.73 and 2.76-2.81 (1H, m, α-C*H* of Phe), 3.69 and 3.71 (3H, s,1 x OC$H_3$), 3.76 and 3.77 (3H, s, 1 x OC$H_3$), 4.41-4.48 and 4.49-4.57 (1H, m, β-C*H* of Phe), 4.79-4.87(1H, m, α-C*H* of Asp), [5.29 and 5.33 (1H, d, J 4.0 Hz, N*H*)], 7.27-7.40 (6H, Ar*H* and N*H*).

Synthesis of Boc-L-Asp(OH)-Z-ΔPhe-OMe (**4**):

DMAP (0.11 equiv, 0.022 g, 0.18 mmol) and $Boc_2O$ (1,1 equiv, 0.376 g, 1.72 mmol) were added to a solution of Boc-L-Asp(Me)-D,L-Phe(β-OH)-OMe (0.6712 g, 1.58 mmol) in dry MeCN (10 mL, 1 M) under rapid stirring at rt. The mixture was monitored by $^1$H NMR and stirred at rt until all the starting material was consumed (typically 5 h). N,N,N′,N′-tetramethylguadinine (4 % in volume, 0.40 mL) was added. The mixture was stirred at rt and monitored by $^1$H NMR until all the intermediate was consumed. Concentration under reduced pressure gave a residue that was partitioned between EtOAc (50 mL) and KHSO4 (1 M, 30 mL). After separation of the phases, the organic phase was washed with KHSO4 (1 M, 2 x 60 mL), NaHCO3 (1 M, 2 x 60 mL) and brine (2 x 60 mL) and then dried with MgSO4. Filtration followed by removal of the solvent afforded Boc-L-Asp(OH)-Z-ΔPhe-OMe (0.358 g, 57%). $^1$H NMR (400 MHz, DMSO-$d_6$) δ: 1.42 (9H, s, OC(C$H_3$)$_3$, 2.84 (1H, dd, J 18.0 Hz, 6.4 Hz, β-C$H_A$$H_B$ of Asp), 3.16 (1H, dd, J 18.0 Hz, 9.2 Hz, β-C$H_A$C$H_B$ of

Asp), 3.75 (3H, s, $CO_2CH_3$), 4.47 (1H, dd, J 16.4 Hz, 7.2 Hz, $\alpha$-C*H* of Asp), 7.32-7.52 (5H, Ar*H* and β-C*H* of ΔPhe), 7.71-7.80 (2H, m, Ar*H* and N*H*), 7.96 (1H, s, N*H* of ΔPhe).

Synthesis of H-Asp(OMe)-Z-ΔPhe-OMe.HCl (**5**):

Boc-L-Asp(OH)-Z-ΔPhe-OMe (0.358 g, 0.88 mmol) was dissolved in TFA (3.0 mL) and the reaction mixture was stirred at room temperature for 1 hour. The TFA was then removed under reduced pressure. Traces of residual TFA were removed by the addition of $CHCl_3$ (3 x 10 mL) followed by removal under reduced pressure, affording H-L-Asp(OMe)-Z-ΔPhe-OMe•HCl as a brown oil. $^1H$ NMR (400 MHz, DMSO-$d_6$) δ: 2.95 (1H, dd, J 15.3 Hz, 5.8 Hz, β-$CH_ACH_B$ of Asp), 3.04 (1H, dd, J 17.2 Hz, 4.0 Hz, β-$CH_ACH_B$ of Asp), 3.68 (3H, s, $OCH_3$ ), 3.70 (3H, s, $OCH_3$ ), 4.33 (1H, dd, J 7.6 Hz, 4.4 Hz $\alpha$-C*H* of Asp), 7.29-7.47 (5H, m, Ar*H* and β-C*H* of ΔPhe and NH), 7.61-7.72 (2H, m, Ar*H*), 8.40 (3H, br s, $H_3N^+$).

Synthesis of Cbz-L-Lys(Cbz)-D,L-Asp(OMe)-Z-ΔPhe-OMe (**6**):

H-Asp(OMe)-Z-ΔPhe-OMe.HCl (0.358 g, 0.85 mmol) was dissolved in MeCN (8 mL) and cooled to 0 °C. Cbz-L-Lys(Cbz)-OH (1.00 equiv, 0.351 g, 0.85 mmol), triethylamine (3 equiv, 0.40 mL, 2.55 mmol) and HBTU (1.1 equiv, 0.353 g, 0.93 mmol) were added sequentially, with 2 min between each addition, and then the mixture was stirred at rt overnight. The solvent was removed under reduced pressure to afford a residue that was partitioned between EtOAc (50 mL) and $KHSO_4$ (50 mL, 1 M). After separation of the phases, the organic layer was thoroughly washed with $KHSO_4$ (1 M, 2 x 50 mL), $NaHCO_3$ (1 M, 2 x 50 mL) and brine (2 x 50 mL) and then dried with $MgSO_4$. Filtration followed by removal of the solvent under reduced pressure afforded Cbz-L-Lys(Cbz)-D,L-Asp(OMe)-Z-ΔPhe-OMe as a white solid (0.446g, 75%). $^1H$ NMR (400 MHz, DMSO-$d_6$) δ: 1.13-1.40 (4H, m, γ-$CH_2$ and δ-$CH_2$ of Lys), 1.45-1.69 (2H, m,β-$CH_2$ of Lys), 2.62-2.73 (1H, m, β-$CH_AH_B$ of Asp), 2.81 (1H, dd, J 36.0 Hz, 6.0 Hz, β-$CH_ACH_B$ of Asp), 2.90-3.02 (2H, m, ε-$CH_2$ of Lys), 3.60 (3H, s, $OCH_3$), 3.68 (3H, s, OCH3), 3.91-4.02 (1H, m, $\alpha$-C*H* of Lys), 4.59-4.71 (1H, m, $\alpha$-C*H* of Asp), 4.98 (2H, s, 1 x $CH_2$ of Cbz), 4.99 (4H, s, 1 x $CH_2$ of Cbz), 7.15-7.49 (16H, Ar*H* and β-C*H* of ΔPhe and 2 x N*H*), 7.60-7.69 (2H, m, Ar*H*), 8.34 (1H, d, J 8.0 Hz, 1 x N*H*),[8.89 and 8.91 (1H, s, N*H* of ΔPhe)]. $^{13}C$ NMR (100.6 MHz, DMSO-$d_6$, δ): 22.6 ($CH_2$, γ-$CH_2$ of Lys), 29.0 ($CH_2$, δ-$CH_2$ of Lys), 31.5 ($CH_2$, β-$CH_2$ of Lys), 36.7 ($CH_2$, β-$CH_2$ of Asp), 40.18 ($CH_2$, ε-$CH_2$ of Lys), 48.59 (CH, $\alpha$-CH of Asp), 52.1 ($CH_3$, 1 x $OCH_3$); 52.8 ($CH_3$, 1 x $OCH_3$), 54.4 (CH, $\alpha$-CH of Lys), 65.0 ($CH_2$, 1 x $CH_2$ of Cbz), 65.3 ($CH_2$, 1 x $CH_2$ of Cbz), 126.0 (CH, Ar), 127.71 (CH, Ar), 128.3 (CH, Ar), 128.5 (CH, Ar), 128.6 (CH, Ar), 129.4 (CH, Ar), 129.9 (CH, Ar), 131.3 (CH, β-CH of ΔPhe), 133.22 (C, $\alpha$-*C* of ΔPhe), 133.24 (C, Ar), 137.0 (C, Ar), 137.2 (C, Ar), 156.06 (C, 2 x *C*=O), 165.3 (C, *C*=O); 169.1 (C,*C*=O); 171.3.3 (C, *C*=O), 171.9 (C, *C*=O).

Synthesis of Boc-L-Lys(Boc)-OH:

L-Lysine monohydrate (2.00g, 11.0 mmol) was dissolved in 1,4-dioxane (65 mL) and then a solution of NaOH 1.0 M (32.7 mL, 33.0 mmol) was added. The solution was left to stir for 10 minutes before $Boc_2O$ (2.00 equiv, 4.5 g, 22.0 mmol) was added. The mixture was then stirred at rt overnight. The 1,4-dioxane solvent was removed under reduced pressure and the mixture was acidified with $KHSO_4$ (1.0 M) until pH 2-3. The aqueous phase was extracted with ethyl acetate (3 x 50 mL) and the organic phase dried with $MgSO_4$. Removal of the solvent under reduced pressure afforded Boc-L-Lys(Boc)-OH as a transparent oil (2.27 g, 60%). $^1H$ NMR (400 MHz, DMSO-$d_6$) δ: 1.19-1.40 (4H, m, γ-$CH_2$ and δ-$CH_2$), 1.42-1.69 (2H, m, β-$CH_2$), 2.81-2.90 (2H, m, ε-$CH_2$), 3.77-3.87 (1H, m, $\alpha$-C*H*), 6.74 (1H, t, J = 18.0 Hz, δ-N*H*), 6.92 (1H, d, J 8.0 Hz, $\alpha$-C*H*), 12.37 (1H, br s, $CO_2H$ of Lys).

Synthesis of Boc-L-Lys(Boc)-D,L-Asp(OMe)-Z-ΔPhe-OMe (**7**):

H-L-Asp(OMe)-Z-ΔPhe-OMe•HCl (0.346g, 0.82 mmol) was dissolved in MeCN (8 mL) and cooled to 0 °C. Boc-L-Lys(Boc)-OH (1.00 equiv, 0.274 g, 0.82 mmol), triethylamine (3 equiv, 0.4 mL, 2.46 mmol) and HBTU (1.1 equiv, 0.342 g, 0.90 mmol) were added sequentially, with 2 min between each addition, and then the mixture was stirred at rt overnight. The solvent was removed under reduced pressure to afford a residue that was partitioned between EtOAc (50 mL) and $KHSO_4$ (50 mL, 1 M). After separation of the phases, the organic layer was thoroughly washed with $KHSO_4$ (1 M, 2 x 50 mL), $NaHCO_3$ (1 M, 2 x 50 mL) and brine (2 x 50 mL) and then dried with $MgSO_4$. Filtration followed by removal of the solvent under reduced pressure afforded Boc-L-Lys(Boc)-D,L-Asp(OMe)-Z-ΔPhe-OMe as a white solid (0.450g, 87%). $^1$H NMR (400 MHz, DMSO-d$_6$) δ:[1.35 and 1.36 (18H, s, OC(C*H*$_3$)], 1.15-1.40 (4H, m, γ-C*H*$_2$ and δ-C*H*$_2$ of Lys), 1.35 (9H, s, 1 x OC(C*H*$_3$)$_3$), 1.36 (9H, s, 1 x OC(C*H*$_3$)$_3$), 1.43-1.64 (2H, m, β-C*H*$_2$ of Lys), 2.61-2.90 (2H, m, β-C*H*$_2$ of Asp), 2.72-2.91 (2H, m, ε-C*H*$_2$ of Lys), 3.60 (3H, s, 1 x OC*H*$_3$), 3.69 (3H, s, 1 x OC*H*$_3$), 3.81-3.95 (1H, m, α-C*H* of Lys); 4.68-4.81 (1H, m, α-C*H* Asp); 6.72 (1H, s, 1 x N*H*); 7.21-7.42 (5H, m, Ar*H* and β-C*H* of ΔPhe and 1x N*H*); 7.60-7.69 (2H, m, Ar*H*), 8.20 (1H, d, J 7.6 Hz, N*H*), [9.48 and 9.61 (1H, s, N*H* of ΔPhe)].

Synthesis of H-L-Lys-D,L-Asp(OMe)-Z-ΔPhe-OMe.2TFA :

Boc-L-Lys(Boc)-D,L-Asp(OMe)-Z-ΔPhe-OMe (0.4501 g, 0.72 mmol) was dissolved in TFA (2.0 mL) and the reaction mixture was stirred at room temperature for 1 hour. The TFA was then removed under reduced pressure. Traces of residue TFA were removed by the addition of $CHCl_3$ (3 x 10 mL) followed by removal under reduced pressure, affording H-L-Lys-D,L-Asp(OMe)-Z-ΔPhe-OMe.2TFA as a brown oil. $^1$H NMR (400 MHz, DMSO-d$_6$) δ: 1.22-1.58 (4H, m, γ-C*H*$_2$ and δ-C*H*$_2$ of Lys), 1.62-1.79 (2H, m, β-C*H*$_2$ of Lys), 2.61-2.72 (2H, m, ε-C*H*$_2$ of Lys), 2.80-2.91 (2H, m, β-C*H*$_2$ of Asp), 3.61 and 3.64 (3H, s, OC*H*$_3$)], 3.81-3.95 (1H, m, α-C*H* of Lys), 4.79-4.90 (1H, m, α-C*H* Asp), 7.15-7.46 (5H, m, Ar*H* and 1x *H*$_3$N$^+$), 7.61-7.86 (2H, m, Ar*H* and β-C*H* of ΔPhe), 8.11-8.29 (2H, m, Ar*H*), 8.86 and 8.94 (3H, d, J=7.2 Hz, 1 x *H*$_3$N$^+$); [9.84 and 9.94 (1H, s, N*H* of ΔPhe)].

Synthesis of Naph-L-Lys(Naph)-D,L-Asp(OMe)-Z-ΔPhe-OMe.2TFA (**8**):

H-L-Lys-D,L-Asp(OMe)-Z-ΔPhe-OMe.TFA (0.4567g, 0.86 mmol) was dissolved in MeCN (8 mL) and cooled to 0 °C. 2-(Naphth-2-yl)-acetic acid (2.00 equiv, 0.320 g, 1.72 mmol), triethylamine (3 equiv, 0.4 mL, 2.58 mmol) and HBTU (1.1 equiv, 0.357 g, 0.94 mmol) were added sequentially, with 2 min between each addition, and then the mixture was stirred at rt overnight. The solvent was removed under reduced pressure to afford a residue that was partitioned between EtOAc (50 mL) and $KHSO_4$ (50 mL, 1 M). After separation of the phases, the organic layer was thoroughly washed with $KHSO_4$ (1 M, 2 x 50 mL), $NaHCO_3$ (1 M, 2 x 50 mL) and brine (2 x 50 mL) and then dried with $MgSO_4$. Filtration followed by removal of the solvent under reduced pressure afforded Naph-L-Lys(Naph)-D,L-Asp(OMe)-Z-ΔPhe-OMe as a white solid (0.210 g, 33%). $^1$H NMR (400 MHz DMSO-d$_6$) δ: 1.16-1.43 (4H, m, γ-C*H*$_2$ and δ-C*H*$_2$ of Lys), 1.43-1.72 (2H, m, β-C*H*$_2$ of Lys), 2.63 (1H, m, β-C*H*$_A$H$_B$ of Asp), 2.81 (1H, m, β-CH$_A$C*H*$_B$ of Asp), 2.90-3.03 (2H, m, ε-C*H*$_2$ of Lys), 3.51-3.62 (4H, m, 2 x C*H*$_2$ of Naph), 3.57 (3H, s, 1 x OC*H*$_3$ ), 3.65 (3H, s, 1 x OC*H*$_3$), 4.20-4.31 (1H, m, α-C*H* of Lys), 4.68-4.82 (1H, m, α-C*H* of Asp), 7.22-7.50 (10H, m, Ar*H* and β-C*H* of ΔPhe), 7.56-7.7.87 (11H, m, Ar*H* and 2 x N*H*), 7.99-8.07 (1H, m, Ar*H* ), 8.38 (1H, d, J = 8.0 Hz, N*H*), 8.59 (1H, d, J = 8.0 Hz, N*H*), [9.58 and 9.61 (1H, s, N*H* of ΔPhe)]. $^{13}$C NMR (100.6 MHz, DMSO-d$_6$, δ): 22.6 (CH$_2$, γ-CH$_2$ of Lys), 28.7 (CH$_2$, δ-CH$_2$ of Lys), 31.9 (CH$_2$, β-CH$_2$ of Lys), 38.2 (CH$_2$, β-CH$_2$ of Asp),

38.6 ($CH_2$, ε-$CH_2$ of Lys), 42.1 ($CH_2$, 1 x *C*$H_2$ of Naph), 42.5 ($CH_2$, 1 x *C*$H_2$ of Naph), 48.5 (CH, *α*-*C*H of Asp), 52.0 ($CH_3$, 1 x $OCH_3$), 52.2 (CH, *α*-*C*H of Lys), 52.8 ($CH_3$, 1 x $OCH_3$), 125.4 (CH, Ar), 126.01 (CH, Ar), 126.04 (CH, Ar), 127.2 (CH, Ar), 127.30 (CH, Ar), 127.36 (CH, Ar), 127.4 (CH, Ar), 127.51 (CH, Ar), 127.56 (CH, Ar), 127.60 (CH, Ar), 127.65 (CH, Ar), 128.4 (CH, Ar), 128.5 (CH, Ar), 128.6 (CH, Ar), 129.1 (C, Ar), 129.8 (CH, Ar), 129.9 (CH, Ar), 130.1 (CH, Ar), 131.3 (CH, β-*C*H of ΔPhe), 131.7 (C, Ar), 132.9 (C, Ar), 134.1 (C, Ar), 134.2 (C, Ar), 169.8 (C, *C*=O), 170.0 (C, *C*=O), 171.41 (C, *C*=O), 171.45 (C, *C*=O).
